# Increases in Future AR Count and Size: Overview of the ARTMIP Tier 2 CMIP5/6 Experiment

**DOI:** 10.1029/2021JD036013

**Published:** 2022-03-21

**Authors:** T. A. O’Brien, M. F. Wehner, A. E. Payne, C. A. Shields, J. J. Rutz, L.‐R. Leung, F. M. Ralph, A. Collow, I. Gorodetskaya, B. Guan, J. M. Lora, E. McClenny, K. M. Nardi, A. M. Ramos, R. Tomé, C. Sarangi, E. J. Shearer, P. A. Ullrich, C. Zarzycki, B. Loring, H. Huang, H. A. Inda‐Díaz, A. M. Rhoades, Y. Zhou

**Affiliations:** ^1^ Department of Earth and Atmospheric Sciences Indiana University Bloomington IN USA; ^2^ Climate and Ecosystem Sciences Division Lawrence Berkeley National Laboratory Berkeley CA USA; ^3^ Computational Research Division Lawrence Berkeley National Laboratory Berkeley CA USA; ^4^ Department of Earth and Space Sciences University of Michigan Ann Arbor MI USA; ^5^ National Center for Atmospheric Research Boulder CO USA; ^6^ National Weather Service, Western Region Headquarters Science and Technology Infusion Division Salt Lake City UT USA; ^7^ Atmospheric Sciences and Global Change Division Pacific Northwest National Laboratory Richland WA USA; ^8^ Center for Western Weather and Water Extremes Scripps Institution of Oceanography University of California, San Diego La Jolla CA USA; ^9^ Universities Space Research Association Columbia MD USA; ^10^ Global Modeling and Assimilation Office NASA Goddard Space Flight Center Greenbelt MD USA; ^11^ Now at University of Maryland Baltimore County Baltimore MD USA; ^12^ Centre for Environmental and Marine Studies Department of Physics University of Aveiro Aveiro Portugal; ^13^ Joint Institute for Regional Earth System Science and Engineering University of California, Los Angeles Los Angeles CA USA; ^14^ Department of Earth and Planetary Sciences Yale University New Haven CT USA; ^15^ Department of Land, Air and Water Resources University of California, Davis Davis CA USA; ^16^ Department of Meteorology and Atmospheric Science Pennsylvania State University University Park PA USA; ^17^ Instituto Dom Luiz (IDL) Faculdade de Ciências Universidade de Lisboa Lisboa Portugal; ^18^ Department of Civil Engineering Indian Institute of Technology Madras Chennai India; ^19^ Center for Hydrometeorology and Remote Sensing University of California, Irvine Irvine CA USA

**Keywords:** atmospheric river, CMIP, ARTMIP, climate change, extreme precipitation

## Abstract

The Atmospheric River (AR) Tracking Method Intercomparison Project (ARTMIP) is a community effort to systematically assess how the uncertainties from AR detectors (ARDTs) impact our scientific understanding of ARs. This study describes the ARTMIP Tier 2 experimental design and initial results using the Coupled Model Intercomparison Project (CMIP) Phases 5 and 6 multi‐model ensembles. We show that AR statistics from a given ARDT in CMIP5/6 historical simulations compare remarkably well with the MERRA‐2 reanalysis. In CMIP5/6 future simulations, most ARDTs project a global increase in AR frequency, counts, and sizes, especially along the western coastlines of the Pacific and Atlantic oceans. We find that the choice of ARDT is the dominant contributor to the uncertainty in projected AR frequency when compared with model choice. These results imply that new projects investigating future changes in ARs should explicitly consider ARDT uncertainty as a core part of the experimental design.

## Key Points

- Uncertainty associated with atmospheric river (AR) definition dominates model uncertainty for projections of Pacific and Atlantic landfalling ARs
- Most AR detection algorithms show an increase in AR frequency in future simulations
- AR statistics in CMIP 5‐and‐6 models compare remarkably well with reanalysis

## 1 Introduction

Over the past 40 years, research on atmospheric rivers (ARs), filamentary bands of intense water vapor transport that were known as tropical cloud plumes in earlier literature, has increasingly demonstrated their importance for cloud and precipitation variability (Iskenderian, 1995; Kiladis & Weickmann, 1992; Kuhnel, 1989; Lau & Chan, 1988; McGuirk et al., 1987; Rasmusson & Arkin, 1993; Thepenir & Cruette, 1981), the global hydrological cycle (Newell et al., 1992; Ralph et al., 2017; Zhu & Newell, 1998) and regional energy and water cycles (Dettinger et al., 2011; Gershunov et al., 2017; Gimeno et al., 2016; Neiman, Ralph, Wick, Kuo, et al., 2008; Newell & Zhu, 1994; Ralph et al., 2005; Shields, Rosenbloom, et al., 2019). ARs are a main source of precipitation and are frequently associated with hydroclimatological impacts in the midlatitude western margins of North America (Guan et al., 2010; Huang et al., 2021; Leung & Qian, 2009; Neiman et al., 2013, 2002; Neiman, Ralph, Wick, Kuo, et al., 2008; Ralph et al., 2013, 2005, 2004; Rutz et al., 2014; Warner et al., 2012), South America (Gimeno et al., 2016; Viale & Nuñez, 2011), Europe (Gimeno et al., 2016; Lavers & Villarini, 2013; Lavers et al., 2012; Ramos et al., 2015; Stohl et al., 2008), and South Africa (Blamey et al., 2018; Ramos et al., 2019). AR impacts on surface heat and water mass balance in polar regions are increasingly evident (Gorodetskaya et al., 2014; Mattingly et al., 2020; Newell & Zhu, 1994; Wille et al., 2019, 2021). Increased understanding of ARs has led to improvements in flood forecasting (Lavers, Pappenberger, et al., 2016; Lavers, Waliser, et al., 2016) and in communication of flood‐related risks when intense ARs are imminent (Ralph, Rutz, et al., 2019).

Numerous recent studies have analyzed ARs in future climate scenarios (e.g., Warner et al., 2015; Lavers et al., 2015; Gao et al., 2015, 2016; Shields & Kiehl, 2016b, 2016a; Polade et al., 2017; Espinoza et al., 2018; Gershunov et al., 2019; Rhoades, Jones, Srivastava, et al., 2020; Rhoades et al., 2021) (see Payne et al. (2020) and references therein). Payne et al. (2020) reviews the related studies over the past 10 years and shows that (a) studies generally agree that global increases in atmospheric moisture will increase the intensity of ARs, and that (b) there is wide uncertainty in the results conveyed in the literature, especially in areas outside the well‐studied U.S. west coast. Existing studies generally agree that the frequency and intensity of ARs will increase, and some studies indicate poleward shifts of the AR tracks (Shearer et al., 2020; Sousa et al., 2020). Gershunov et al. (2019) show that intermodel differences in future projections of precipitation are much lower when considering precipitation due to ARs than those when considering changes in bulk precipitation. Given that precipitation is produced by a variety of meteorological phenomena, and that there is no guarantee that the relative proportions of precipitation from various phenomena are the same in models as they are in observations, Gershunov et al. (2019) highlight the importance in using a phenomenon‐focused study of precipitation in future climate simulations.

Essentially all of the studies of ARs and future climate (and past climate, e.g., Kiehl et al., 2018; Lora et al., 2017; Menemenlis et al., 2021; Skinner et al., 2020) rely on objective, quantitative methods to discriminate ARs from the background: AR detectors (ARDTs). At present, ARs have a qualitative definition (Ralph et al., 2018), which leaves researchers with the task of implementing a quantitative definition of ARs in specific ARDTs. ARDTs typically consist of a set of heuristic rules (e.g., thresholds and filters) that focus on identifying anomalously high moisture or moisture transport that occurs in contiguous, filamentary structures. The design of ARDTs is guided by understanding gained through decades of observational and model studies (Bao et al., 2006; Browning & Pardoe, 1973; Lackmann & Gyakum, 1999; McGuirk et al., 1987; Neiman et al., 2002; Neiman, Ralph, Wick, Kuo, et al., 2008; Neiman, Ralph, Wick, Lundquist, & Dettinger, 2008; Newell et al., 1992; Ralph et al., 2005, 2004; Waliser et al., 2012; Zhu & Newell, 1998). The number of ARDT algorithms has grown with the number of ARDT studies over the past decade, with new ARDTs often being developed for specialized purposes: For example, ARDTs for understanding the global hydrological cycle (Guan & Waliser, 2015; Zhu & Newell, 1998), observed hydrometeorological extremes (Neiman, Ralph, Wick, Lundquist, & Dettinger, 2008; Rutz et al., 2014), the cryosphere (Gorodetskaya et al., 2014; Wille et al., 2021), and regional hydroclimate variability (Gershunov et al., 2017). Even though ARDTs are often initially designed with different purposes in mind, Payne et al. (2020) demonstrate that there is overlap in what they are ultimately used to study. The community has recently started to recognize that uncertainty associated with the numerical definition of ARs may have important implications for our understanding of ARs and their changes in a future warmer world (Guan et al., 2018; Huning et al., 2017; Lora et al., 2020; Newman et al., 2012; O’Brien, Payne, et al., 2020; O’Brien, Risser, et al., 2020; Ralph, Wilson, et al., 2019; Rutz et al., 2019; Shields et al., 2018; Shields, Rosenbloom, et al., 2019; Shields, Rutz, et al., 2019)

The Atmospheric River Tracking Method Intercomparison Project (ARTMIP) was launched by members of the AR research community in order to systematically assess the impact of this uncertainty on our scientific understanding (Shields et al., 2018). The First ARTMIP Workshop (Shields, Rutz, et al., 2019) defined a multi‐tier experimental design focusing on uncertainty in the observational record (Tier 1; Rutz et al., 2019), and uncertainty in AR variability and change (Tier 2). Two Tier 2 experiments were launched at the Second ARTMIP Workshop (Shields, Rutz, et al., 2019): the Tier 2 C20C+ experiment and the Tier 2 CMIP5/6 experiment. Both experiments are designed to elucidate the effect of uncertainty associated with ARDTs on our understanding of ARs, with the former focusing on uncertainty in regional impacts in a single high‐resolution global model, and the latter focusing on the relative roles of model and ARDT‐associated uncertainty. A third Tier 2 experiment was launched at the Third ARTMIP Workshop: the Tier 2 Reanalysis experiment, which aims to understand how differences across reanalyses compare with differences across ARDTs. This manuscript overviews the Tier 2 CMIP5/6 experiment.

## 2 Data and Methods

We use data from the ARTMIP Tier 1 experiment (Rutz et al., 2019; Shields et al., 2018), which provides atmospheric river detections from multiple ARDT algorithms. All Tier 1 ARDTs run on a common set of atmospheric fields (e.g., integrated vapor transport) derived from the Modern‐Era Retrospective analysis for Research and Applications, Version 2 (MERRA‐2; Gelaro et al., 2017). A subset of the Tier 1 algorithms have also been run on the Tier 2 input data set described further on. The subset of algorithms run was determined by the subset of ARTMIP participants who volunteered to run their algorithms on the Tier 2 data set; these algorithms include ARCONNECT_v2 (Shearer et al., 2020), Guan_Waliser_v2 (Guan & Waliser, 2015; Guan et al., 2018), IDL_rel_future and IDL_rel_hist (Blamey et al., 2018; Ramos et al., 2016), Lora_v2 (Lora et al., 2017; Skinner et al., 2020), Mundhenk_v3 (Mundhenk et al., 2016), PNNL_v1 (Hagos et al., 2015), and TECA‐BARD v1.0.1 (O’Brien, Risser, et al., 2020), and Tempest (McClenny et al., 2020; Ullrich & Zarzycki, 2017) (see Table S1 in the Supporting Information S1). Text S4 in the Supporting Information S1 describes why choice of reanalysis unlikely affects the qualitative conclusions of this paper.

For the Tier 2 input data set for ARDTs, we derive integrated water vapor (IWV), and the components of the integrated vapor transport (IVT) vector from outputs from atmosphere‐ocean general circulation models associated with the Coupled Model Intercomparison Project (CMIP) Phases 5 (Taylor et al., 2012) and 6 (Eyring et al., 2016; O’Neill et al., 2016) multi‐model ensembles (hereafter referred to as CMIP5/6 when both ensembles are jointly discussed). We utilize model output from the historical simulations in both CMIP5 and CMIP6, and we utilize output from the representative concentration pathway 8.5 (RCP8.5, CMIP5) and shared socioeconomic pathways 5–8.5 experiments (SSP5‐8.5, CMIP6). We utilize models that provided specific humidity *q* (hus) and wind $\vec{u}$ (ua and va) at 6‐hourly intervals on the native model vertical grid (the 6hrLev table); we further restrict the set of models to those which provide model output from the same ensemble member for both the historical and future (RCP8.5 and SSP5‐8.5) simulations. We chose to focus on models providing data on the native model vertical grid (either sigma or hybrid‐sigma) because this facilitates an accurate calculation of vertical integrals without having to handle below‐ground levels as would be necessary if dealing with model output on isobaric surfaces; this choice simplifies interpretation of inter‐ARDT differences in continental interiors, where such below‐ground levels are common. At the time that the Tier 1 input data set was constructed (in Summer 2019), we were able to access 6 models from CMIP5 (CCSM4, CSIRO‐Mk3‐6, CanESM2, IPSL‐CM5A‐LR, IPSL‐CM5B‐L, and NorESM1‐M) and 3 models from CMIP6 (BCC‐CSM2‐MR, IPSL‐CM6A‐LR, MRI‐ESM2‐0; Boucher et al., 2019; Xin et al., 2019; Yukimoto et al., 2019) that satisfied these constraints (see Table S1 in the Supporting Information S1): 9 models in total and one ensemble member from each model. We focus on the 1981–2010 time period for the historical reference period, and we calculate trends over the 1951–2099 period (some data are missing due to data availability and corruption issues, and years with these issues are not included in calculations; see Text S3 in the Supporting Information S1). Examination of the 1951–2099 timeseries at a variety of locations show that changes in AR frequency are close to linear; therefore the trends presented here can be used to infer discrete changes in AR frequency at arbitrary timeperiods (e.g., mid‐century and end‐of‐century). The models selected represent a range of horizontal resolutions (ranging from approximately 100–300 km), and the RCP8.5 and SSP5‐8.5 scenarios represent aggressive emission trajectories with large amounts of radiative forcing (nominally 8.5 W/m^2^) by end‐of‐century.

The mass‐weighted vertical integrals of water vapor (*ρq*) and water vapor transport ($\rho \vec{u}q$) are calculated from all native model levels in the CMIP5/6 output as:

(1) $$\text{IWV}=-\frac{1}{g}\sum_{k=1}^{N}q_{k}\Delta p_{k}$$

(2) $$\vec{\text{IVT}}=-\frac{1}{g}\langle \sum_{k=1}^{N}u_{k}q_{k}\Delta p_{k},\sum_{k=1}^{N}v_{k}q_{k}\Delta p_{k}\rangle ,$$

where index *k* corresponds to model levels going from the surface (*k* = 1) to the top of the model atmosphere (*k* = *N*), and Δ*p*_*k*_ is the difference in level pressures, estimated at level *k*. The total vapor transport is calculated as the vector magnitude: $\text{IVT}=\left|\vec{\text{IVT}}\right|$.

These ARDTs consist of a mixture of algorithms that detect ARs globally (global algorithms) and algorithms designed for specific regions (regional algorithms); see Table S1 in the Supporting Information S1. We focus most of the analysis in this manuscript on the location of the AR tracks, changes in these tracks, and uncertainty therein. We therefore focus the bulk of the discussion on the global subset of algorithms; the full set of algorithms is discussed in Section 3.3 when comparing the relative magnitudes of uncertainty related to ARDT design and model choice.

### 2.1 Tier 2 CMIP5/6 Experiment Overview

All Tier 2 CMIP5/6 ARDT contributions use the common data set of IWV, IVT, and $\vec{\text{IVT}}$ described in Section 2, which come from 9 models in the CMIP5 and CMIP6 multi‐model ensembles. ARDT outputs are regridded to a common 4° × 5° latitude‐longitude grid. We assess the CMIP5/6 models by comparing annual spatial patterns of AR frequency between the Tier 1 and Tier 2 experiments, for each detection scheme independently, focusing on spatial pattern correlation and spatial variability. Given the 6‐hourly frequency of the data set, we report frequency as ‘equivalent’ AR days, which we define as 0.25 times the total number of timesteps with AR conditions. We provide details about Tier 2‐specific modifications to ARDTs in Text S1 in the Supporting Information S1 and details about missing data in Text S3 in the Supporting Information S1.

Grouping algorithms by the type of criteria applied (relative vs. absolute thresholds) and degree of restrictiveness (magnitude of thresholds employed, number of criteria involved) can reduce the spread associated with ARDTs (Ralph, Wilson, et al., 2019; Rutz et al., 2019). Here, we group ARDTs into three categories, based on their treatment of thresholds: *absolute* (ARCONNECT_v2, PNNL_v1, and Lora_v2), *fixed relative* (Guan_Waliser_v2, IDL_rel_future, IDL_rel_hist, and Mundhenk_v3), and *relative* (Tempest and TECA‐BARD v1.0.1). The categorizations are described and justified in Text S2 in the Supporting Information S1. A key motivation for this categorization is aggregating ARDTs by their sensitivity to thermodynamic changes in IVT, with the assumption that ARDTs employing absolute thresholds to moisture fields will be the most sensitive, and ARDTs employing time‐dependent thresholds will be least sensitive.

## 3 Results

### 3.1 Evaluation of Historical Simulations

**Figure 1:**
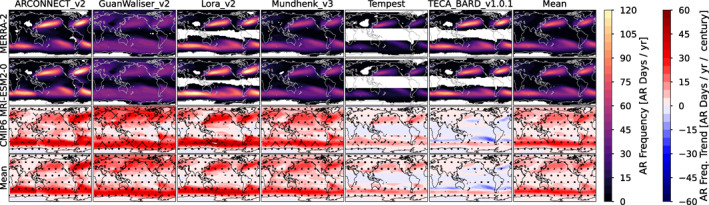
(first and second rows) Maps of atmospheric river (AR) frequency (shown as average number of days with AR conditions) annually for the 1981–2010 period. Each column corresponds to a global AR detection algorithm, and the last column represents the average across all AR detection algorithms. The top row corresponds to AR detections on the Modern‐Era Retrospective analysis for Research and Applications, Version 2 data set (the Tier 1 Atmospheric River Tracking Method Intercomparison Project experiment) and the second and third rows correspond to AR detections on the CMIP6 MRI‐ESM2‐0 simulation. White indicates areas where average AR occurrence is fewer than 1 day. (third and fourth rows) Maps of trends in annual AR frequency in the MRI‐ESM2‐0 simulation (third row) and all models (fourth row), organized by detection algorithm (columns) from 1951 to 2099 (with a few exceptions noted in the text). Trends significant at the 90% level (according to a 2‐sided *t*‐test) are indicated by stippling, and trends significant at the 95% level are indicated by cross‐hatching.

We show maps of annual average AR frequency from the Tier 1 (MERRA‐2) experiments for the 6 global ARDT algorithms in the top row of Figure 1. The ARDTs show broad consistency in the spatial patterns of ARs. All ARDTs identify well‐known AR tracks, with distinct maxima in the midlatitude Pacific and the Atlantic, and with a circumglobal maximum in the Southern Ocean; these AR tracks have been described in papers using multiple ARDTs (e.g., Gimeno et al., 2016; Guan & Waliser, 2015; Lavers et al., 2012; Lora et al., 2020; Zhu & Newell, 1998). The ARDTs also identify significant areas with little or no AR activity: the tropics, northeastern Asia, northeastern South America, tropical and subtropical Africa, the subtropical eastern Pacific (near the cold tongue region), as well as interiors of both polar regions (except for with Guan_Waliser_V2). The ARDTs differ significantly in the relative frequency of AR conditions. Some of the ARDTs identify AR conditions occurring upwards of 120 days per year (approximately one third of the time) in the main AR tracks, and other ARDTs identify AR conditions occurring fewer than 40 days per year. These results are consistent with previous ARDT comparisons, indicating a wide range of restrictiveness across ARDTs (Lora et al., 2020; Ralph, Wilson, et al., 2019; Rutz et al., 2019). The algorithms also differ in the degree to which the AR tracks penetrate inland and the maximum poleward extension of the AR tracks (poleward non‐zero AR boundary), with the Guan_Waliser_v2 algorithm commonly identifying ARs in continental interiors and polar regions, and TECA‐BARD v1.0.1 rarely identifying ARs in continental interiors and polar regions. The average frequency of ARs (the top‐right panel in Figure 1) exhibits a similar spatial pattern to the various ARDTs, with ARs occurring approximately 60 days per year in the core AR track.

Simulated ARs in the Tier 2 CMIP5/6 experiment are remarkably consistent with those in the Tier 1 MERRA‐2 experiment. Results from an arbitrary model–MRI‐ESM‐2‐0 from the CMIP6 multimodel ensemble–are shown in the second row of Figure 1, and a similar plot showing results from all possible model‐ARDT pairs is shown in Figure S1 in the Supporting Information S1. The placement of the AR tracks (and opposing gaps in ARs) are very similar when comparing spatial maps for a given ARDT. The algorithm‐mean AR frequencies (last column) show very little difference between Tier 1 and 2; this is true for all models analyzed (see Figure S1 in the Supporting Information S1).

Each ARDT has idiosyncratic spatial patterns that are expressed in both Tier 1 and Tier 2. This suggests that the spatial pattern maps are an emergent property of each ARDT, and that these spatial patterns are relatively insensitive to significant changes in the representation of the underlying atmospheric dynamics. For example, the diffuse spatial pattern associated with the Guan_Waliser_v2 (GW) algorithm is evident in Tier 1 and in all Tier 2 simulations (Figures S1 and S2 in the Supporting Information S1), and the multi‐model mean for the GW algorithm exhibits a similar spatial pattern. This suggests that there is much more variability in AR frequency across ARDT algorithms than there is across simulations; we quantify this in Section 3.3.

**Figure 2:**
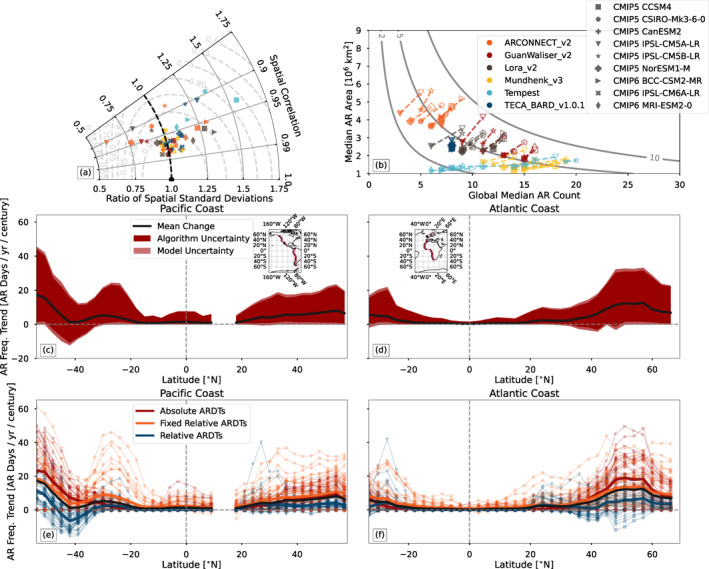
(a) A Taylor diagram comparing the spatial correlation (azimuthal axis) and spatial variability (radial axis) of atmospheric river (AR) frequency between Coupled Model Intercomparison Project Phases 5 and 6 simulations (denoted by different symbols) and the Modern‐Era Retrospective analysis for Research and Applications, Version 2 reanalysis. Colors indicate different AR detection algorithms (legend in panel b). Gray dashed lines show lines of constant skill score (Taylor, 2001). (b) Median AR area versus global median AR count for all available combinations of ARDTs (marker colors) and simulations (marker symbols). Filled symbols indicate calculations performed on the 1981–2010 period of each simulation, and open symbols indicate calculations on the 2070–2099 period (two exceptions noted in Text S3 in the Supporting Information S1). Gray contours show lines of constant fractional areal coverage of ARs (shown as a percentage of Earth's area), calculated as the product of AR area and AR count, divided by Earth's area. (c and d) Trends in AR frequency (black curve) and associated total range of uncertainty (blue and light blue shading) for the west‐facing (c) Pacific coastline and (d) Atlantic coastline. Dark blue shading indicates the portion of uncertainty associated with AR detection and the light blue shading indicates the portion of the spread associated with models (across both CMIP5 and CMIP6). The area of dark blue shading is proportional to $\sigma_{A}^{2}/\sigma_{T}^{2}\cdot \left(\text{max}-\text{min}\right)$, where ‘max’ and ‘min’ are the minimum and maximum trend at each latitude. (e and f) as in (c and d), but showing individual ARDT‐model combinations. Markers indicate simulations (legend in panel b) and colors indicate the ARDT classification. Bold lines indicate the mean trend across the ARDT classification. The inset maps in (c and d) show the Pacific and Atlantic coast masks respectively.

Figure 2a quantitatively shows that CMIP5 and CMIP6 simulations compare well with the MERRA‐2 reanalysis when compared within a single ARDT. Spatial correlation coefficients between the AR frequency maps in individual Tier 2 simulations and the corresponding Tier 1 map are above *r* = 0.95 for most ARDT‐model pairs (32 out of 52 pairs), and the ratio of spatial standard deviations of AR frequency (Tier 2 divided by Tier 1) is between 0.75 and 1.25 for 40 out of 52 ARDT‐model pairs. The Taylor skill scores (Taylor, 2001) are above 0.9 for 37 out of 52 ARDT‐model pairs. Variability exists, with some ARDT‐model pairs reaching as high as *r* ≈ 0.97 and only 5 ARDT‐model pairs with correlation coefficients between 0.8 and 0.9 (and skill scores below 0.85); likewise, one combination (ARCONNECT_v2 and CMIP5 IPSL‐CM5A‐LR) has variability that is too low by approximately 25%, and one combination (Tempest and CMIP5 IPSL‐CM5B‐LR) has variability that is about 50% too high. Overall, this emphasizes the high degree of similarity between simulated ARs and ARs in MERRA‐2, when comparing results using a single ARDT.

Altogether, the various ARDTs portray a similar assessment of model skill, with essentially all of the models analyzed appearing to be ‘fit for purpose’. This is true even for the lowest resolution simulations (e.g., CMIP5 CanESM2 with a nominal 310 km horizontal resolution in the tropics; see Table S1 in the Supporting Information S1), which have some of the highest correlation coefficients. (Note that the AR detection process was performed at the original model resolution, prior to regridding to a common grid for comparison with reanalysis.). A survey of the literature (Gao et al., 2015; Hagos et al., 2015; Shields & Kiehl, 2016b; Guan & Waliser, 2017; Payne et al., 2020; Reid et al., 2020; Rhoades, Jones, O’Brien, et al., 2020) indicates a mix of possible resolution effects, with some indication that the effect of resolution may depend on the experimental setup (e.g., coupled vs. uncoupled; Guan & Waliser, 2017). We hypothesize that resolution effects may depend on the ARDT used; these effects could be studied more systematically by applying multiple ARDTs to the CMIP6 HighResMIP experiment (Haarsma et al., 2016). The ARTMIP community has discussed the possibility of coordinating a Tier 2 Resolution experiment (O’Brien, Payne, et al., 2020) to explore this more systematically.

Results associated with the Tempest algorithm are a somewhat notable exception: five of the models evaluated with Tempest have high spatial variability relative to MERRA‐2, and relatively low spatial correlations. This may be related to some differences in the implementation of Tempest between the Tier 1 and Tier 2 experiments (see Text S1 in the Supporting Information S1).

### 3.2 Projected Changes in AR Frequency, Count, and Size

When the ARDTs are applied to the various future simulations described in Section 2, they project a variety of trends in AR frequency. Figure 1 (third row) shows that most ARDTs applied to the MRI‐ESM2‐0 simulation indicate increases in AR frequency in the main AR tracks. Within each algorithm, the trends from the MRI‐ESM2‐0 simulation are quantitatively and qualitatively similar to trends from other simulations (see Figure S3 in the Supporting Information S1), as indicated by the similarity between the MRI‐ESM2‐0 trends and the multi‐model trends shown in the bottom row of Figure 1. The average trend across all model‐ARDT combinations (lower right panel of Figure 1) likewise indicates an increase in AR frequency in the midlatitude storm tracks, with increases of ∼20 AR days per year per century (an approximate 30% increase). In addition to this increase in AR frequency in the mid‐latitude storm tracks, it is also important to note an increase in the areas with historically rare or close to zero frequency of the ARs, such as southern Asia and Africa, the Arctic Ocean and the Antarctic ice sheet. There are essentially no ocean basins where the model‐ARDT mean indicates a decrease in AR frequency.

The climatological pattern of AR frequency is primarily controlled by changes in AR size, AR occurrence (count), and AR location. Two ARDTs (TECA‐BARD v1.0.1 and to a lesser extent Tempest) suggest poleward shifts in AR location (Figure 1, bottom row, and Figure S3 in the Supporting Information S1), whereas ARCONNECT_v2, GuanWaliser_v2, Lora_v2, and Mundhenk_v3 indicate quasi‐global increases in AR frequency. We discuss why differences in the quantitative definition of ARs may cause different behavior in future climate simulations and its implications in Section 4. We have run the same analysis for seasonal averages for all four seasons, and the seasonal climatology and seasonal trends are similar to the annual average results presented in Figure 1.

We decompose the changes in AR frequency by changes in AR area *A* and AR count *N*; Figure 2b shows the median size of AR objects versus the median number of AR objects counted at any given time. In the historical simulations, the ARDTs appear to cluster along a continuum, with ARDTs typically detecting 5–20 ARs, which is consistent with manual counts of ARs in synoptic maps (Zhu & Newell, 1998; O’Brien, Risser, et al., 2020). Tempest is a notable exception, with AR counts ranging from 20–50. In order to aid in interpreting the continuum along which the ARDTs lay in Figure 2b, we add lines of constant global area *A*_⊕_ percentage (calculated as 100*%* ⋅ *A* ⋅ *N*/*A*_⊕_). These show that algorithms typically detect ARs such that approximately 5% of the Earth's surface is covered in AR objects in the historical simulations. Therefore, we can interpret the relative location of ARDTs in Figure 2b as an indicator of the relative spatial coherence of AR objects: ARDTs on the left detect few, large AR objects and ARDTs on the right detect many small AR objects. This grouping along lines of constant global area fraction is an emergent collective behavior of the ARDTs, and we speculate that it is associated with the tuning process for each algorithm. AR coherence might make a useful measure for objective grouping of AR results in future ARTMIP studies.

Figure 2b shows that four of the ARDTs (except Tempest and TECA‐BARD v1.0.1) tend to detect more ARs and larger ARs in the future simulations. These changes result in increases in the global area coverage of AR objects: changing from ∼5% global area to ∼7% global area. The global count of AR objects does not change in the TECA‐BARD v1.0.1 algorithm, though there are slight increases in AR area in some simulations. In contrast, the Tempest algorithm indicates increases in global AR count, with very little change in AR area.

There is an indication that the resolution of the underlying model may affect the characteristics of detected ARs for some ARDTs. The CMIP6 BCC‐CSM2‐MR, CMIP6 MRI‐ESM2‐0, and CMIP5 CCSM4 simulations–which are the three highest resolution simulations analyzed (Table S1 in the Supporting Information S1)–tend to occur on the right side of each ARDT cluster: ARs in these simulations are systematically less coherent. However, the model resolution does not appear to affect the climate change signal evident in Figure 2b. Further, the CMIP5/6 simulations analyzed here do not attempt to control for model resolution; the CMIP6 HighResMIP experiment (Haarsma et al., 2016) could provide a way to examine resolution effects more systematically.

### 3.3 Sources of Uncertainty in End‐of‐Century Projections of ARs

The results in Figure 1 indicate that there may be substantial uncertainty in future AR frequency associated with choice of ARDT. Further, it is not clear from the spatial maps in Figure 1 whether the trends in AR frequency evident over the ocean (e.g., the decrease in the southeastern Atlantic) extend to the coastal areas where AR presence matters for western‐coastal water cycles and hydrometeorological impacts. We quantify these changes and their uncertainty in Figures 2c and 2d, which show the mean trend in AR frequency for the Pacific (Figure 2c) and Atlantic west coasts (Figure 2d) from 1951 to 2099. Figures 2c and 2d show trends for all ARDTs listed in Table S1 in the Supporting Information S1: both regional and global ARDTs.

Figures 2c and 2d show that coastal areas in both the Pacific and Atlantic show increasing trends in AR frequency (+1–10 AR days per year per century in the midlatitudes), and the full spread of the red and light red shading in Figures 2c and 2d show the full range of trends from all ARDTs and all models. There are two areas where TECA‐BARD v1.0.1 indicates weakly decreasing trends (Figure S3 in the Supporting Information S1 shows the trends by model and by algorithm): southern Chile, near 40°S, and near the entrance of the Mediterranean Sea from 35°N to almost 60°N, which spans the Mediterranean, Iberian Peninsula and British Isles. It is noteworthy that this decrease is compensated by an increase in AR frequency poleward of these regions, indicating a poleward shift in the AR frequency. Otherwise all model‐ARDT combinations indicate increasing trends in landfalling AR frequency for both Pacific and Atlantic ARs in both hemispheres.

Large uncertainty appears in the magnitude of the trends, which ranges from just below 0 days/yr/century to over 60 days/year/century, depending on location. There are two main components of uncertainty in these trends: uncertainty associated with choice of model simulation, and uncertainty associated with choice of ARDT. We decompose the uncertainty as $\sigma_{T}^{2}\approx \sigma_{A}^{2}+\sigma_{M}^{2}$, where $\sigma_{T}^{2}$ is the total variance, $\sigma_{A}^{2}$ is the variance across ARDTs of each ARDT's multi‐model mean, and $\sigma_{M}^{2}$ is the variance across models for each model's multi‐ARDT mean. These variances can equivalently be viewed as the variance down the rightmost column in Figure S3 in the Supporting Information S1 ($\sigma_{M}^{2}$) and the variance across the bottommost row in Figure S3 in the Supporting Information S1 ($\sigma_{A}^{2}$), (excluding the multi‐model/multi‐ARDT mean in the bottom right corner of Figure S3 in the Supporting Information S1 and excluding trends from MERRA‐2).

This decomposition shows that uncertainty associated with choice of ARDT accounts for most of the spread in the climate change signal across all latitudes in both the Pacific and Atlantic coasts. In essence, uncertainty associated with the numerical definition of ARs dominates the combined uncertainty associated with choice of model and choice of model epoch (CMIP5 vs. CMIP6). As shown in Figures 1 and 2, comparison against reanalysis shows that most ARDT‐model pairs perform well when compared with reanalysis, so this measure of model skill does not provide a way to reduce the uncertainty, since all ARDTs perform equivalently well on average. If there were a standard against which to rank ARDTs, it might be possible to utilize ARDT‐weighting approaches to narrow the spread; but such a standard currently does not exist, and so such a weighting approach is not possible.

The spread in the number of detected ARs accounts for some of the spread in trends. If the trends in Figures 2c–2f are normalized by the number of ARs detected, the relative magnitude of the ARDT‐related uncertainty drops, though it is still large: above 50% of the total spread in the midlatitudes. (Note that this quantity is ill‐defined in regions, such as the tropics, where few or no ARs are detected.) As suggested by O’Brien, Risser, et al. (2020), this suggests that constraining the total number of ARs is of central importance to reducing uncertainty about AR variability and change.

## 4 Discussion and Conclusions

While there have been studies examining future changes in ARs (e.g., Payne et al., 2020) and studies examining uncertainty related to choice of ARDT (e.g., Rutz et al., 2019), no existing study has attempted to quantify the attribution of ARDT uncertainty for climate change by evaluating model uncertainty versus ARDT uncertainty. The ARTMIP Tier2 CMIP5/6 experiment provides a unique opportunity for such a study. The results from this experiment show that most ARDTs project an increase in AR frequency, with mean trends of approximately +1–10 AR days/year per century along the western coastlines of North America, South America, Southern Africa, and Europe (Figures 2c and 2d). These changes are relatively large, given that the AR frequency in coastal regions is typically between 5–30 AR days per year, though this depends strongly on geographic region and the ARDT used (Figure 1). However, there is considerable spread in the magnitude, with some ARDT‐model combinations indicating negative trends (southern Chile and the European west coast from the Iberian Peninsula to the British Isles) with a clear AR shift poleward and other ARDT‐model combinations indicating positive trends of ARs in all regions with a magnitude up to ∼60 AR days per century. Care must be taken when making general statements about the sign of AR frequency/size/count trends, since this work shows that the sign and magnitude of the trends are linked to choices that ARDT designers make when translating the qualitative AMS definition into a quantitative definition. Specific statements can be made if one settles on a narrow quantitative definition, as is typically done when seeking answers to questions about processes or impacts related to ARs (e.g., orographic precipitation, ice sheet melt, or process drivers).

Globally, all ARDTs indicate either an increase in the total number of ARs, an increase in the areal extent of ARs, or both (Figure 2b). In the historical simulations, the AR area versus size relationship for all ARDTs approximately falls along a line of constant global coverage, with ARDTs in the historical simulations detecting ARs that cover approximately 5% of the global area. This number is somewhat smaller than the 10% global area indicated by Zhu and Newell (1998), which is likely because we are considering the total global coverage, including the tropics, rather than the fraction of zonal circumference in the midlatitudes. It is nevertheless qualitatively consistent in the sense that areas of anomalously high moisture transport occupy a small fraction of the global area. The global areal coverage increases in the future simulations to some degree in all ARDT algorithms, with most indicating a several percent increase in the areal extent of ARs due to increases in both AR size and count.

These results further show that future changes in AR frequency can qualitatively differ depending on the type of ARDT used. We aggregate trends by AR classification (see Sections 2 and Text S2 in the Supporting Information S1) in Figures 2e and 2f. This aggregation shows that use of any absolute thresholds (*absolute ARDTs*) and time‐independent relative thresholds (*fixed relative ARDTs*) tend to produce increases in AR frequency, whereas use of time‐dependent relative thresholds (*relative ARDTs*) tend to produce patterns more indicative of a poleward shift. *Absolute ARDTs* and *fixed relative ARDTs*, with thresholds that do not change in time, would be expected to increase the frequency of exceedance of regions above the historical thresholds: more detected AR days in a warmer climate. Such ARDTs are designed to detect increases in occurrence of regions with high IVT, which are important for AR impacts. In contrast, *relative ARDTs* (e.g., TECA‐BARD v1.0.1) are designed to only account for dynamical–rather than thermodynamical–changes in ARs.

**Figure 3:**
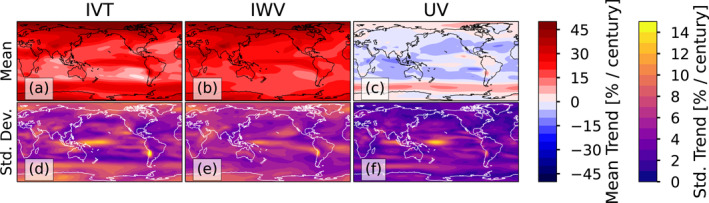
Trends in IVT, IWV, and UV ≡ IVT/IWV among the CMIP5/6 models, calculated from approximately 1950–2100. Panels (a–c) show the mean trend, and panels (d–f) show the standard deviation of the trends. Trends for each model are shown in Figure S4 in the Supporting Information S1.

To illustrate the thermodynamic and dynamic changes in IVT, Figures 3a–3c shows the model‐mean trend in IVT, IWV and the moisture‐weighted wind UV ≡ IVT/IWV (it can readily be demonstrated that *UV* represents the vertically averaged wind, weighted by the specific humidity at each height). The model spread in these trends are shown in Figures 3d–3f (Figure S4 in the Supporting Information S1 shows the trends for each model). Both the IVT and IWV fields increase at a rate of 20%–40% per century in the model simulations, whereas the UV field has much smaller changes: decreases in wind of 5%–15%/century in most of the tropics and midlatitudes and increases of similar magnitude in the polar regions. Because IVT is the product of IWV and UV, the fractional trend in IVT can be decomposed into a sum of the fractional trends in each quantity:

$$\frac{1}{\text{IVT}}\frac{\partial \text{IVT}}{\partial t}=\frac{1}{\text{IWV}}\frac{\partial \text{IWV}}{\partial t}+\frac{1}{\text{UV}}\frac{\partial UV}{\partial t}.$$

The similarity of the IVT and IWV trend magnitudes implies that most of the trend in IVT is due to the thermodynamic component: the increase in atmospheric water vapor content due to Clausius‐Clapeyron (CC) scaling. In contrast, the dynamic change is more indicative of a poleward shift in the magnitude of moisture‐transporting winds. It is worth noting that the results presented in Figure 3 are independent of ARDT, though they do help explain some of the differences across ARDTs.

The literature documents two major modes of AR change associated with climate change: (a) A quasi‐global increase in IVT associated with CC scaling (thermodynamic; Payne et al., 2020), and (b) a poleward shift in ARs (dynamic; Payne et al., 2020) associated with the poleward shift in the midlatitude storm tracks (Chang et al., 2012). Poleward shift patterns appear to co‐exist to some extent with quasi‐global increases in AR frequency in some simulations (e.g., the CMIP5 CSIRO‐MK3‐6‐0 simulation; see Figure S3 in the Supporting Information S1) for all ARDTs. We argue that *absolute ARDTs* and *fixed relative ARDTs* are more sensitive to thermodynamic changes than *relative ARDTs*. The strongest increase in the *absolute* and *fixed relative ARDTs* compared to *relative ARDTs* explains the sensitivity to ARDT choice especially approaching colder and drier polar regions. The future, much stronger, increase in high latitude temperature associated with polar amplification, compared to other regions, together with hydrological cycle intensification will be more evident in the *absolute* and *fixed relative ARDTs* compared to the *relative ARDTs*.

This categorization of ARDTs does not perfectly explain the spread in trends, as Tempest and TECA‐BARD v1.0.1 trends in Figure 1 are qualitatively different; as such the mean trends for the *relative ARDTs* in Figures 2e and 2f should be interpreted with caution. We hypothesize that they differ due to how the two methods identify relative peaks in the IVT field: Tempest uses the Laplacian to find local ridges in the IVT field, whereas the percentile‐based approach in TECA‐BARD v1.0.1 seeks out the relatively highest IVT locations in each timestep. It is possible that Tempest identifies relatively small, weak ARs that TECA‐BARD v1.0.1 misses because they are weak enough to fall below its relative threshold. If this is the case, it could imply that the contrasting regions, where Tempest shows an increase and TECA‐BARD v1.0.1 shows a decrease, are associated with an increase in the occurrence of relatively weak ARs that TECA‐BARD v1.0.1 misses. This is worth studying in a future paper.

It is worth noting here that trend patterns in the MERRA‐2 reanalysis are similar across ARDTs (Figure S3 in the Supporting Information S1), with all ARDTs indicating a poleward shift in ARs. This might suggest that the observed poleward shift in the storm tracks (Davis & Rosenlof, 2012; Fyfe, 2003; Lucas et al., 2014; Manney & Hegglin, 2018; Pena‐Ortiz et al., 2013; Tilinina et al., 2013) dominates over quasi‐global increases in IVT in the historical record. This should be investigated further as part of the Tier 2 Reanalysis experiment.

The algorithm‐wise validation of simulated ARs (Figure 2a) shows that models exhibit spatial patterns of AR occurrence similar to those in reanalysis, as evidenced by high Taylor skill scores for spatial correlations and standard deviations. This is a noteworthy result in the context of the ARDT uncertainty shown here. If only one algorithm is used in a study, such validation could give false confidence in the robustness of results. It therefore seems important to explicitly include ARDT uncertainty as part of evaluation of a model's ability to represent ARs, which, relatedly, points to the utility of appropriate ensemble weighting strategies to help reduce such uncertainty (e.g., Massoud et al., 2019). It also highlights the value of AR‐related, but not ARDT‐dependent, evaluations of models (e.g., Payne & Magnusdottir, 2015).

Recent work involving manual identification of ARs by experts (Prabhat et al., 2021; O’Brien, Risser, et al., 2020) suggests that the spread in AR algorithm behavior is linked to differences in opinion about what does and does not constitute an AR. O’Brien, Risser, et al. (2020) show that this spread in subjective opinion projects directly on to quantitative differences in the sign of the correlation coefficient between an El Niño index and global AR count. Such differences in subjective opinion likely also play a role in the quantitative choices made by various ARDT designers. Gimeno et al. (2021) add some discussion concerning the diversity of the different meteorological patterns that can be associated with the qualitative definition of ARs, and there is no guarantee that all so‐called ARs are associated with the same meteorological patterns. Given this spread in expert opinion, and given that there is no agreed‐upon theoretical or numerical definition of what defines an AR, there is presently no way to objectively assess whether one ARDT is better than another.

Somewhat relatedly, the ARTMIP project has established that different AR detectors are designed with different–and equally legitimate–purposes (Ralph, Wilson, et al., 2019; Rutz et al., 2019; Shields et al., 2018). Some ARDTs intentionally choose to discriminate ARs from the background based on absolute thresholds in IVT (e.g., Rutz et al., 2014), since it is well‐established that coastal orographic precipitation is directly linked to IVT magnitude (Neiman et al., 2002; Neiman, Ralph, Wick, Kuo, et al., 2008; Ralph, Rutz, et al., 2019; Ralph et al., 2005, 2004); such a design choice makes it easy to relate ARDT results directly to hydrometeorological impacts. Other algorithms (e.g., Shields & Kiehl, 2016b; O’Brien, Risser, et al., 2020) intentionally use relative thresholds in order to avoid increases in AR detection due to long‐term increases in atmospheric water vapor. Both are valid for the purposes for which they were designed: absolute methods detect areas that will likely lead to hydrometeorological impacts–which will increase in a warmer climate–and relative methods seek to focus on the core of regions associated with anomalous vapor transport.

These results suggest that new projects investigating future changes in the statistics and characteristics of ARs should explicitly consider ARDT uncertainty as a core part of the experimental design. This study makes it clear that ARDT design choices can have a major impact on the results of climate change studies, and with dozens of ARDTs in use (Rutz et al., 2019), the uncertainty associated with their varying methods will not be going away soon. Furthermore, using multiple ARDTs can be advantageous. For example, will an increase in ARs and precipitation result primarily from an increase in IWV or an increase in UV wind? Having ARDTs that weigh these variables differently can help answer these questions. The Bayesian, multi‐ARDT approach of O’Brien, Risser, et al. (2020) can quantify parametric uncertainty associated with a single ARDT, but it is not yet clear how parametric uncertainty compares to structural uncertainty (i.e., choices in what heuristic rules to employ in the ARDT). There are at least four ARDT codes that are now in the public domain (Mundhenk_v1, Guan_Waliser_v2, Tempest, and TECA‐BARD v1.0.1; see https://www.cgd.ucar.edu/projects/artmip/algorithms.html for a full list of ARDTs that have participated in ARTMIP), and we encourage current and future ARDT designers to likewise enter their codes into the public domain in order to facilitate such uncertainty exploration in future studies.

Ralph et al. (2018) provide a concise, qualitative definition of ARs, and this has been a major benefit to the AR research community. They intentionally chose to “leave specifications of how the boundaries of an AR are to be quantified open for future and specialized developments.” The results in this manuscript demonstrate that the choice of how to define AR boundaries–the fundamental job of an ARDT–have a demonstrably large control on the statistics of ARs detected in future climate simulations. These results suggest that the AR research community would further benefit from studies that aim to quantitatively constrain the definition of ARs; for example, with first‐principles analyses that constrain AR properties like size, count, etc. Such constraints could help reduce uncertainty associated with ARDT design choice (and parameter choice), and by extension they could constrain results concerning ARs and future climate change. That said, given that different experiments motivate different ARDT design choices (e.g., absolute vs. relative thresholds), it seems unavoidable that some of this uncertainty is irreducible. It is clear, however, that it is imperative for studies to explore and understand the implications of this uncertainty.

This study focuses on a bulk, global perspective of uncertainty associated with ARDTs and simulations in the Tier 2 CMIP5/6 experiment. There are many other types of more detailed analyses that others could take on. For example, this study has not considered the temporal characteristics of ARs, since relatively few existing ARDTs track ARs as they propagate in time; a recent study by Zhou et al. (2021) uses a common temporal tracking algorithm on multiple ARDTs, and such an approach could be applied to the Tier 2 data set. We encourage others in the research community to utilize this data set for research on future ARs and climate change (see data availability statement in Acknowledgments). In particular, it seems valuable to revisit past studies of ARs and future climate change in the context of ARDT uncertainty. Payne et al. (2020) review the numerous results concerning the future of ARs that have appeared in the literature in the last decade. There are almost as many ARDTs as there are such results, which makes intercomparison of the results challenging. The Tier 2 CMIP5/6 data set provides a way to revisit many–if not all–of these previous results within a uniform experimental framework.

Prior to ARTMIP, it was assumed that the various ARDTs in the literature were simply different methods of looking at the same dynamical phenomenon. Recent papers associated with ARTMIP show that that is true for strong ARs (with high IVT, e.g., Rutz et al., 2019; Lora et al., 2020), but that there is disagreement among the various ARDTs for weaker ARs. Further, Zhang et al. (2019) show that approximately 20% of ARs are not associated with a nearby extratropical cyclone (under their ARDT criteria), suggesting that this subset of ARs may have a different dynamical origin. This raises some questions that remain unanswered. *Are some ARDTs simply missing ARs that other ARDTs are identifying, or is there more than one type of dynamical phenomenon that produces AR‐like objects*; *are some ARDTs more sensitive to one dynamical phenomenon and others are more sensitive to another*; and *if there are multiple dynamical causes of ARs, do they have different spatiotemporal responses to climate change?* These questions are likely answerable with the datasets that have been produced by the ARTMIP project.

In summary, this initial analysis of the Tier 2 CMIP5/6 experiment shows that most ARDTs and simulations indicate an increasing trend in AR frequency, size, and number in future simulations with strong radiative forcing. It also shows the critical importance of understanding the implications of uncertainty for AR‐related research. Finally, this paper introduces the publicly available Tier 2 CMIP5/6 data set, which may be a valuable resource for answering fundamental questions about ARs and about ARs and climate change.

## Supporting information

Supporting Information S1

## Data Availability

ARTMIP Tier 2 CMIP5/6 catalogs can be found on the Climate Data Gateway: https://doi.org/10.26024/s4p7ߚpf13.
