# Supplementary material for: Increases in Future AR Count and Size: Overview of the ARTMIP Tier 2 CMIP5/6 Experiment
Source: J Geophys Res Atmos. 2022 Mar 21;127(6):e2021JD036013. doi: 10.1029/2021JD036013 (PMC9285484; doi:10.1029/2021JD036013)
Supplement: Supplementary file 1 — Supporting Information S1 [file JGRD-127-0-s001.pdf]

Supporting Information for "Increases in Future AR Count and Size: Overview of the ARTMIP Tier 2 CMIP5/6 Experiment"

T. A. O'Brien[1,2] *, M. F. Wehner[3] and A. E. Payne[4] and C. A. Shields[5] and J. J. Rutz[6] and L.-R. Leung[7] and F. M. Ralph[8] and A. Collow[9,10] and I. Gorodetskaya[11] and B. Guan[12] and J. M. Lora[13] and E. McClenny[14] and K. M. Nardi[15] and A. M. Ramos[16] and R. Tomé[17] and C. Sarangi[7,18] and E. J. Shearer[17] and P. A. Ullrich[14] and C. Zarzycki[15] and B. Loring[3] and H. Huang[2] and H. A. Inda-Díaz[14,2] and A. M. Rhoades[2] and Y. Zhou[2]

[1]Dept. of Earth and Atmospheric Sciences, Indiana University, Bloomington, IN, USA

[2]Climate and Ecosystem Sciences Division, Lawrence Berkeley National Laboratory, Berkeley, CA, USA

[3]Computational Research Division, Lawrence Berkeley National Laboratory, Berkeley, CA, USA

[4]Dept. of Earth and Space Sciences, University of Michigan, Ann Arbor, MI, USA

[5]National Center for Atmospheric Research, Boulder, CO, USA

[6]National Weather Service, Western Region Headquarters, Science and Technology Infusion Division, Salt Lake City, UT, USA

[7]Atmospheric Sciences and Global Change Division, Pacific Northwest National Laboratory, Richland, WA, USA

[8]Center for Western Weather and Water Extremes, Scripps Institution of Oceanography, University of California, San Diego, La Jolla, CA, USA

[9]Universities Space Research Association, Columbia, MD, USA

[10]Global Modeling and Assimilation Office, NASA Goddard Space Flight Center, Greenbelt, MD, USA

[11]Centre for Environmental and Marine Studies, Dept. of Physics, University of Aveiro, Portugal

[12]Joint Institute for Regional Earth System Science and Engineering, University of California, Los Angeles, CA, USA

[13]Dept. of Earth and Planetary Sciences, Yale University, New Haven, CT, USA

[14]Dept. of Land, Air and Water Resources, University of California, Davis, Davis, CA, USA

[15]Dept. of Meteorology and Atmospheric Science, Penn State University, University Park, PA, USA

[16]Instituto Dom Luiz (IDL), Faculdade de Ciências, Universidade de Lisboa, Lisboa, Portugal

[17]Center for Hydrometeorology and Remote Sensing, University of California, Irvine, Irvine, CA, USA

[18]Department of Civil Engineering, Indian Institute of Technology Madras, India

Contents of this file

1. Text S1 to S4
2. Figures S1 to S5
3. Table S1

Introduction

This supplementatal information provides additional useful details on ARDTs, their treatment of thresholds, and our grouping of ARDTs into categories. The supplemental figures expand on figures in the main text to show all ARDT-simulation combinations.

Text S1.

Treatment of Thresholds

Corresponding author: T. A. O'Brien, Department of Earth and Atmospheric Sciences, Indiana University, 1001 E. 10th Street, Bloomington, IN 47408, USA (obrienta@iu.edu)

*Dept. of Earth and Atmospheric Science,
1001 E. 10th St, Bloomington, IN, 47408

We document here choices/specializations (if any) that ARDT contributors made in running their ARDTs on the Tier 2 CMIP5/6 simulations

- `ARCONNECT_v2`: only uses absolute threshold; no Tier 2-specific decisions needed
- `Guan_Waliser_v2`: uses 85th percentile from the historical simulation
- `IDL_rel_future`: uses 85th percentile calculated from the future simulation
- `IDL_rel_hist`: uses 85th percentile calculated from the historical simulation
- `Lora_v2`: uses a time-and-latitude dependent IVT threshold that asymptotes to 225 kg/m/s at the poles; the time/latitude dependence of the threshold is a function of the 30-day running mean and a zonal average of IWV, so no Tier 2-specific decisions are needed
- `Mundhenk_v3`: calculates the mean and seasonal cycle of IVT based on the historical simulation and removes this to determine the IVT anomaly relative to the historical period
- `PNNL_v1`: only uses absolute threshold; no Tier 2-specific decisions needed
- `TECA_BARD_v1.0.1`: uses threshold relative to spatial map of IVT at a given time; no Tier 2-specific decisions needed

The `Mundhenk_v3` algorithm differs from prior published versions (i.e., `Mundhenk_v1`, `Mundhenk_v2`) in its more reliable detection of AR objects that cross the boundary of the dataset's spatial domain.

The `Tempest` ARDT uses an absolute threshold for the laplacian of IVT. The Tier 1 version also utilized an absolute threshold of 250 kg/m/s of IVT, but it was later determined that this threshold had no effect on the ARDT results because regions that satisfied the Laplacian threshold also satisfied the IVT threshold. The minimum latitude for ARs was raised to 20°, from 15°, to filter easterly waves. The stencil radius and

magnitude used for the Laplacian depends on the model grid, and this is held constant for the historical and future simulations.

Discussions with the `Tempest` contributing scientists indicate that the algorithm may benefit from further tuning of their method when applied to moderately low resolution data, and efforts are underway to provide a second version of their contribution to Tier 2. Such discoveries and improvements are a benefit of intercomparison projects.

Text S2.

Classification of ARDTs

Building on Rutz et al. (2019), we classify the Tier 2 CMIP5/6 ARDTs into three groups, based on their treatment of thresholds: *absolute*, *fixed relative*, and *relative*. These classifications are indicated as *abs.*, *fix. rel.*, and *rel.* in Table S1. A key motivation for this categorization is aggregating ARDTs by their sensitivity to thermodynamic changes in IVT, with the assumption that ARDTs employing absolute thresholds to moisture fields will be the most sensitive, and ARDTs employing time-dependent thresholds will be least sensitive.

Absolute ARDTs: We define *absolute ARDTs* as utilizing any fixed thresholds (e.g., in IVT) for discriminating ARs from the background. `ARCONNECT_v2` and `PNNL_v1` unambiguously fit in this category. `Lora_v2` uses an IVT threshold that varies with latitude and time, and the threshold asymptotes to 250 kg/m/s at mid-to-high latitudes (the threshold increases toward infinity approaching the tropics). This design effectively imposes an absolute threshold of at least 250 kg/m/s. Because of this, we classify `Lora_v2` as an *absolute ARDT*, while recognizing that this is not a perfect categorization.

Fixed relative ARDTs: We define *fixed relative ARDTs* as those that employ relative thresholds that do not vary with time. For example, `Guan_Waliser_v2` calculates the 85th percentile of IVT from the historical simulations and discriminates ARs from the background where IVT is greater than the local, historical 85th percentile; hence the threshold used in the `Guan_Waliser_v2` algorithm does not change in time. The `IDL_rel_hist` and `IDL_rel_future` ARDTs use a similar approach and are therefore also categorized as *fixed relative ARDTs*. `Mundhenk_v3` calculates IVT anomalies relative to the historical period and identifies ARs that are above the 94th percentile of the historical simulation, so it also fits unambiguously in the *fixed relative* category.

Relative ARDTs: We define *relative ARDTs* as those that employ relative thresholds that vary with time. `TECA_BARD_v1.01` unambiguously fits into this category, since ARs are identified where IVT is above a fixed percentile of IVT, where the percentile is calculated in space (in contrast to time, e.g., for `Guan_Waliser_v2`). `Tempest` uses an absolute threshold applied to the Laplacian of the IVT field, which might warrant its classification as an absolute ARDT. However, the use of the Laplacian removes the mean of the IVT field; therefore `Tempest` identifies areas of IVT that are high relative to nearby areas of IVT at the same timestep. We therefore classify `Tempest` as a *relative ARDT*.

Text S3.

Details on Missing Data All ARDTs detect ARs for the 1951-2099 period for the combined historical and future simulations for each CMIP5/6 model. We analyze output from the entire 1951-2099 timeperiod. There are some exceptions to this: output from the CMIP6 IPSL-CM6A-LR SSP5-8.5 simulation are only available through 2049, there are data corruption issues for the year 2006 in the CMIP5 CSIRO-Mk3-6-0 simulation, and

there are data corruption issues for the year 2095-2099 for the `TECA_BARD_v1.01` output applied to the CMIP5 IPSL-CM5B-LR simulation. Years with data corruption issues are marked as missing, and trends and climatologies are only calculated considering non-missing data. The `Guan_Waliser_v2` algorithm did not supply ARDT catalogues for the NorESM1-M and BCC-CSM2-MR simulations due to technical issues at the time.

Text S4.

Comment on the Tier 2 Reanalysis Experiment

The tiered structure of the ARTMIP experiments requires that all participants contribute to the Tier 1 experiment; by design, all ARDTs participating in the Tier 2 CMIP5/6 experiment also have been run on MERRA-2 as part of the Tier 1 experiment. A separate ARTMIP Tier 2 experiment is currently underway, comparing ARDT results applied to different reanalyses. The set of ARDTs participating in the Tier 2 Reanalysis experiment is not identical to the set participating in this Tier 2 CMIP5/6 experiment, so use of multiple reanalyses is not possible for ARDTs. For the sake of uniformity in the experimental approach, we use only the MERRA-2 reanalysis. Preliminary analysis of the Tier 2 Reanalysis experiment (not shown) indicates that the uncertainties associated with choice of reanalysis are small compared to the uncertainties discussed in this paper, and it is therefore unlikely that use of a different reanalysis would change the qualitative conclusions of this paper.

Table S1. (left) ARDT algorithms, and associated metadata, that contributed to the Tier 2 CMIP5/6 experiment. ARDT classifications ('Class.') are described in Text S2. (right) Details of CMIP5/6 models used in the Tier 2 experiment.

ARDTs				Models			
Algorithm ID	Contrib.	Class.	Region	MIP Era	Model Name	Inst.	~Res. [km]
ARCONNECT_v2	Shearer	abs.	Global	CMIP5	CCSM4	NCAR	120
GuanWaliser_v2	Guan	fix. rel.	Global	CMIP5	CSIRO-Mk3-6	CSIRO	207
IDL_rel_future	Ramos	fix. rel.	W. Europe, S. Africa	CMIP5	CanESM2	CCCMA	310
IDL_rel_hist	Ramos	fix. rel.	W. Europe, S. Africa	CMIP5	IPSL-CM5A-LR	IPSL	296
Lora_v2	Lora	abs.	Global	CMIP5	IPSL-CM5B-LR	IPSL	296
Mundhenk_v3	Nardi	fix. rel.	Global	CMIP5	NorESM1-M	NCC	242
PNNL_v1	Sarangi	abs.	W. U.S.	CMIP6	BCC-CSM2-MR	BCC	124
Tempest	McClenny	rel.	Global	CMIP6	IPSL-CM6A-LR	IPSL	198
TECA_BARD_v1.01	O'Brien	rel.	Global	CMIP6	MRI-ESM2-0	MRI	124

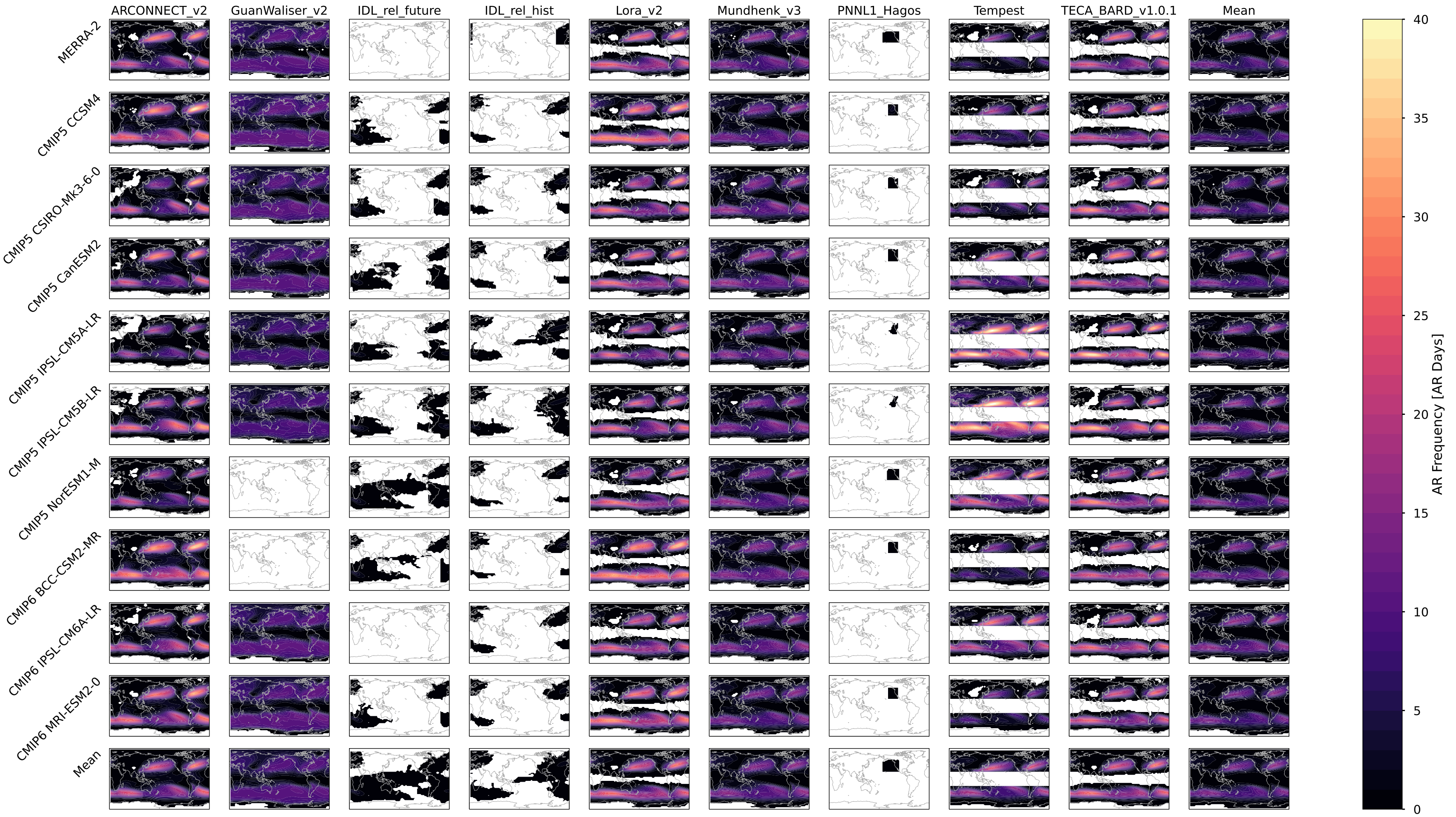

Figure S1. Maps of AR frequency (AR days per year) from 1981-2010. Columns correspond to ARs detected by specific ARDTs, and rows correspond to input datasets (MERRA-2 for the first row and CMIP5/6 for other rows). The rightmost column shows the multi-ARDT mean frequency for each model. The bottom row shows the multi-model mean for each ARDT (excluding MERRA-2 from the mean). The bottom right panel shows the multi-model, multi-ARDT mean frequency (excluding MERRA-2 from the mean).

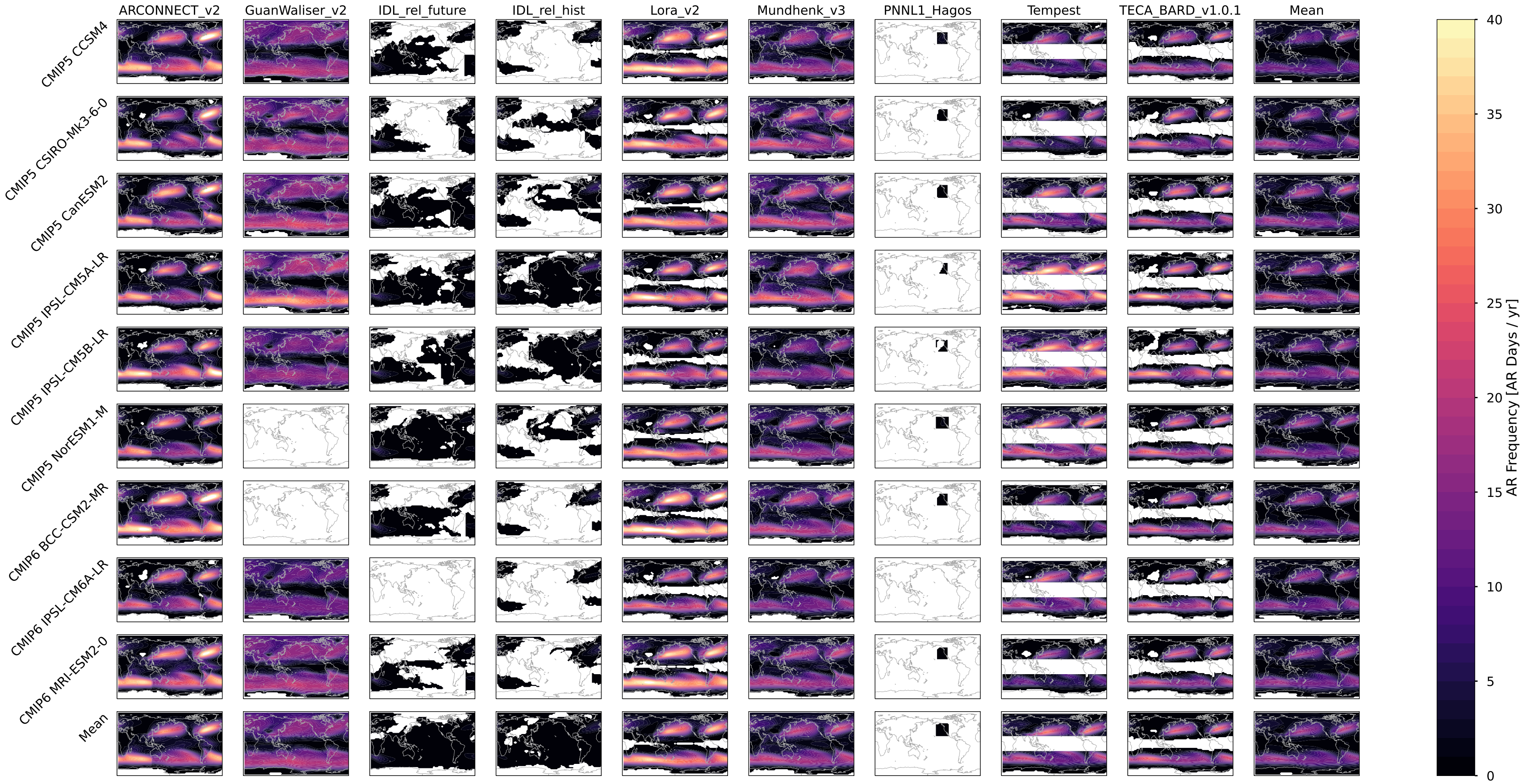

Figure S2. Maps of AR frequency (AR days per year) from 2070-2099. Columns correspond to ARs detected by specific ARDTs, and rows correspond to CMIP5/6 models. The rightmost column shows the multi-ARDT mean frequency for each model. The bottom row shows the multi-model mean for each ARDT. The bottom right panel shows the multi-model, multi-ARDT mean. The CMIP6 IPSL-CM6A-LR row shows the frequency from the last 30 years of available simulation data (2020-2049).

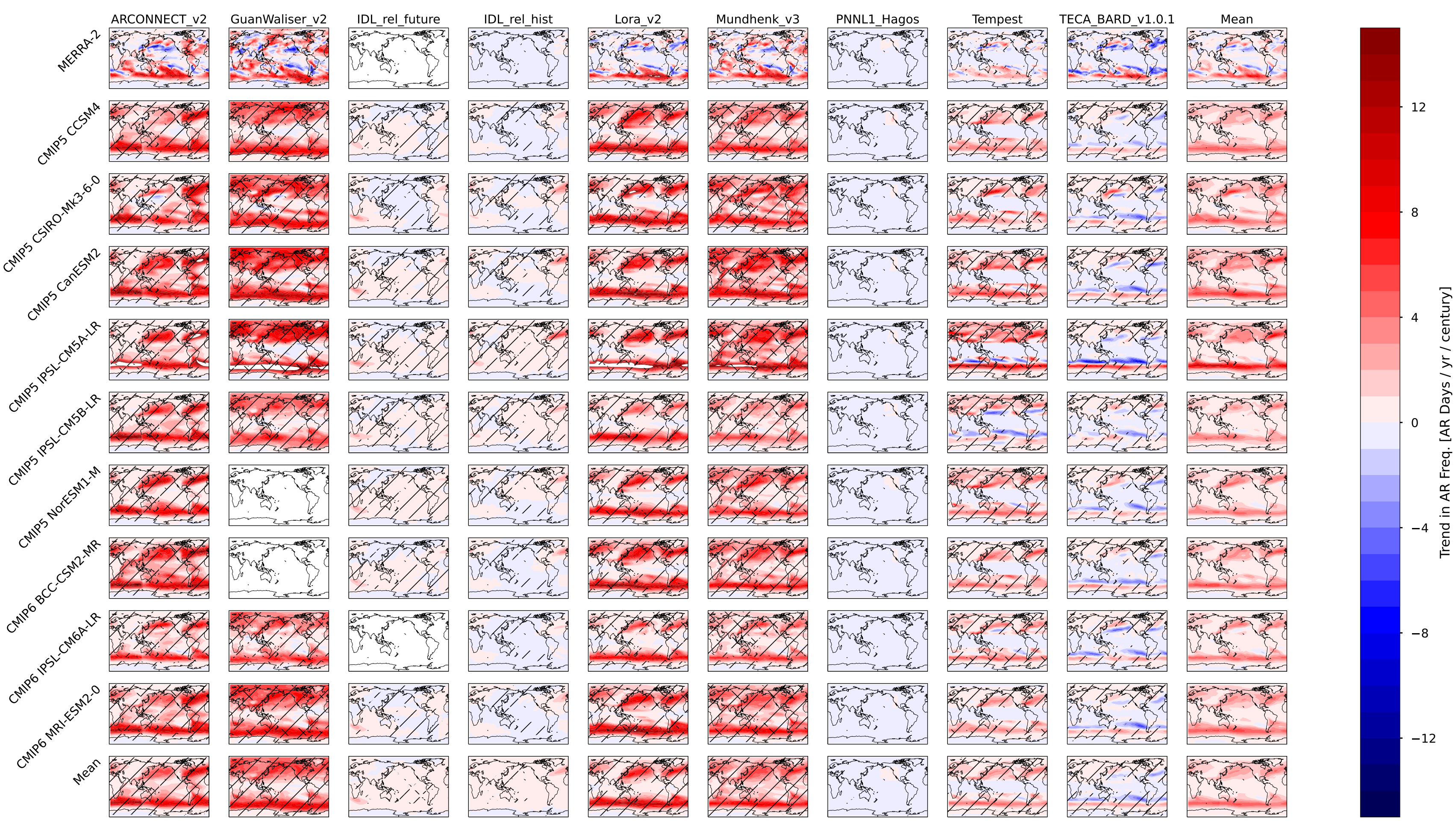

Figure S3. Maps of trends in AR frequency (AR days per year per century) from 1981-2099 (from 1981-2017 for MERRA2). Columns correspond to ARs detected by specific ARDTs, and rows correspond to input datasets (MERRA-2 for the first row and CMIP5/6 for other rows). The rightmost column shows the multi-ARDT mean trend for each model. The bottom row shows the multi-model mean for each ARDT (excluding MERRA-2 from the mean). The bottom right panel shows the multi-model, multi-ARDT mean frequency (excluding MERRA-2 from the mean). Trends for CMIP6 IPSL-CM6A-LR are calculated through 2049.

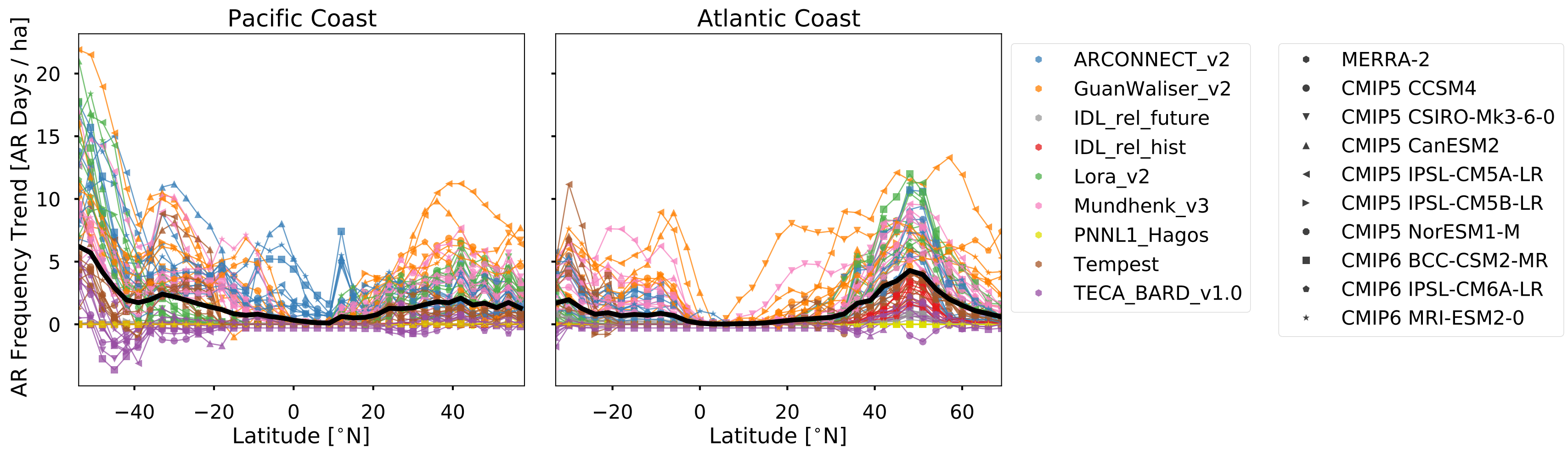

Figure S4. Trends in AR frequency (AR days per year per century) from 1981-2099 for western coastlines along the (a) Pacific and (b) Atlantic oceans. Colors correspond to ARDTs and markers correspond to simulations.

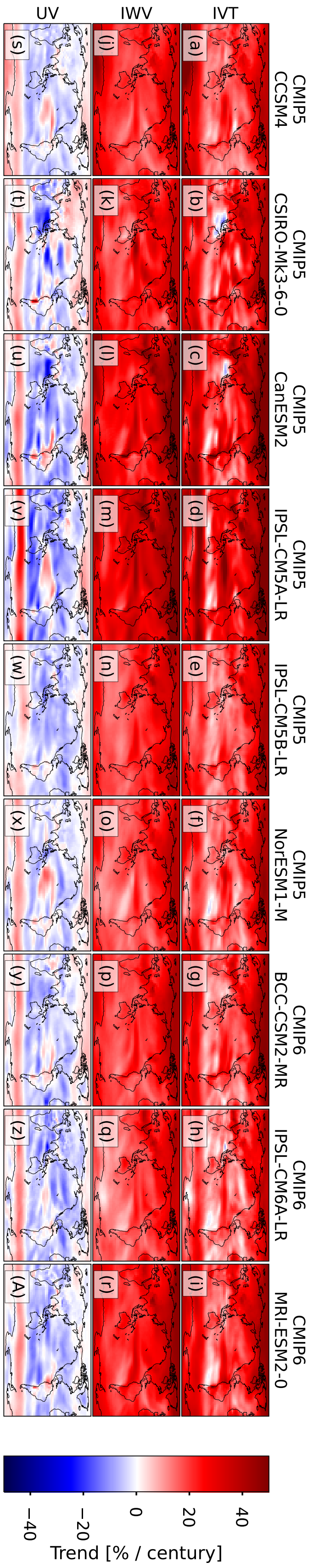

Figure S5. Trends in IVT (a–i), IWV (j–r), and UV≡IVT/IWV (s–A) for each of the CMIP5/6 models, calculated from approximately 1951–2099.
